# The MOSAIC study - comparison of the Maudsley Model of Treatment for Adults with Anorexia Nervosa (MANTRA) with Specialist Supportive Clinical Management (SSCM) in outpatients with anorexia nervosa or eating disorder not otherwise specified, anorexia nervosa type: study protocol for a randomized controlled trial

**DOI:** 10.1186/1745-6215-14-160

**Published:** 2013-05-30

**Authors:** Ulrike Schmidt, Beth Renwick, Anna Lose, Martha Kenyon, Hannah DeJong, Hannah Broadbent, Rachel Loomes, Charlotte Watson, Shreena Ghelani, Lucy Serpell, Lorna Richards, Eric Johnson-Sabine, Nicky Boughton, Linette Whitehead, Jennifer Beecham, Janet Treasure, Sabine Landau

**Affiliations:** 1 PO59, Section of Eating Disorders, Department of Psychological Medicine, Institute of Psychiatry, King’s College London, De Crespigny Park, London SE5 8AF, UK; 2 Oxford Adult Eating Disorder Service, Cotswold House, Warneford Hospital, Oxford, UK; 3 MHRN North London Hub, 20 Eastbourne Terrace, London, UK; 4 Hope Wing, Porters Avenue Health Centre, Dagenham, Essex, UK; 5 The Phoenix Wing, St Ann’s Hospital, Tottenham, London, UK; 6 Personal Social Services Research Unit, London School of Economics and Political Science, London, UK; 7 Personal Social Services Research Unit, University of Kent, Canterbury, UK; 8 Department of Biostatistics, Institute of Psychiatry, Kings College London, London, UK; 9 Research Department of Clinical, Educational & Health Psychology, University College London, London, UK

**Keywords:** Anorexia nervosa, Eating disorder not otherwise specified, Outpatient treatment, Randomized controlled trial, Cost effectiveness

## Abstract

**Background:**

Anorexia nervosa (AN) is a biologically based serious mental disorder with high levels of mortality and disability, physical and psychological morbidity and impaired quality of life. AN is one of the leading causes of disease burden in terms of years of life lost through death or disability in young women. Psychotherapeutic interventions are the treatment of choice for AN, but the results of psychotherapy depend critically on the stage of the illness. The treatment response in adults with a chronic form of the illness is poor and drop-out from treatment is high. Despite the seriousness of the disorder the evidence-base for psychological treatment of adults with AN is extremely limited and there is no leading treatment. There is therefore an urgent need to develop more effective treatments for adults with AN. The aim of the Maudsley Outpatient Study of Treatments for Anorexia Nervosa and Related Conditions (MOSAIC) is to evaluate the efficacy and cost effectiveness of two outpatient treatments for adults with AN, Specialist Supportive Clinical Management (SSCM) and the Maudsley Model of Treatment for Adults with Anorexia Nervosa (MANTRA).

**Methods/Design:**

138 patients meeting the inclusion criteria are randomly assigned to one of the two treatment groups (MANTRA or SSCM). All participants receive 20 once-weekly individual therapy sessions (with 10 extra weekly sessions for those who are severely ill) and four follow-up sessions with monthly spacing thereafter. There is also optional access to a dietician and extra sessions involving a family member or a close other. Body weight, eating disorder- related symptoms, neurocognitive and psychosocial measures, and service use data are measured during the course of treatment and across a one year follow up period. The primary outcome measure is body mass index (BMI) taken at twelve months after randomization.

**Discussion:**

This multi-center study provides a large sample size, broad inclusion criteria and a follow-up period. However, the study has to contend with difficulties directly related to running a large multi-center randomized controlled trial and the psychopathology of AN. These issues are discussed.

**Trial Registration:**

Current Controlled Trials ISRCTN67720902 - A Maudsley outpatient study of treatments for anorexia nervosa and related conditions.

## Background

Anorexia nervosa (AN) is a biologically based serious mental disorder with high levels of mortality and disability, physical and psychological morbidity and impaired quality of life [1]. Cognitive and emotional functioning are impaired [2-4], and motivation may be compromised due to the disorder being highly valued [5], making engagement in treatment difficult.

AN is one of the leading causes of disease burden in terms of years of life lost through death or disability in young women [6], and the cost per case of AN is at least equal to that of schizophrenia [7,8]. Compared to other mental disorders, AN has the highest proportion of hospital admissions with a length of stay over 90 days (26.8%) and the longest median length of stay (36 days) [9]. In one study of adolescents with AN, the annual service cost was found to be approximately 17,000 GBP [10], in adults with more chronic disorders this is likely to be higher. The family members are usually the main carers and report similar burden to carers of people with psychosis [11]. A systematic review [12] of the costs of eating disorders identified two cost-of-illness studies, both of which underestimated the costs because of important omitted cost items. The review concludes that the costs of AN are likely to be substantial. A recent report detailed the cost of eating disorders to healthcare and wider society [13]. It estimated a total cost for England, per year of 1.25 billion GBP. This figure includes costs to the NHS and private healthcare, the human costs and the cost of lost output. The report specifically estimated the cost to healthcare being over 80 million GBP. Most of this can be attributed to the cost of AN.

Psychotherapeutic interventions are the treatment of choice for AN, but the result of psychotherapy depends critically on the stage of the illness. Whilst response to psychological treatment (usually family-based) is excellent in adolescents with a short duration of AN [14], the treatment response in adults with a more chronic form of the illness is much less positive and drop-out from treatment is high [15]. The evidence-base for psychological treatment of adults with AN is extremely limited. Several small trials have tested a range of therapies, including cognitive-behavioral therapy (CBT), interpersonal therapy (IPT), cognitive analytical therapy, and family therapy, but as yet no one treatment has been found to be the best in terms of efficacy [16,17]. One small trial [18] found specialist supportive clinical management (SSCM) superior on a range of outcomes compared to CBT and IPT at end of treatment. Due to the superiority of SSCM compared with the other active treatments this intervention has been selected as the comparison treatment in this randomized controlled trial (RCT). The urgent need to develop more effective treatments for adults with AN has been highlighted [16,17].

One of the key factors responsible for the relative lack of efficacy of treatments for adults with AN is that most of these have been adapted from those for other disorders and are neither tailored sufficiently to the characteristics and needs of people with AN nor focused on how the disorder is maintained. To remedy this problem we have developed a specific maintenance model and treatment approach for AN [5], the Maudsley Model of Treatment for Adults with AN (MANTRA). Our treatment model is novel in several respects: (a) it is empirically-based, drawing on and incorporating recent neuropsychological, social cognitive and personality trait research in AN, (b) it includes *both* intra- and interpersonal maintaining factors, and proposes strategies for addressing these and, (c) it is modularized with a clear hierarchy of procedures and tailored to the needs of the individual. Findings from pilot studies demonstrate the acceptability and efficacy of this treatment intervention among the AN patient group [19,20].

### Aims

The main aim of this study is to compare the efficacy, cost and cost-effectiveness of MANTRA in adult outpatients with AN with that of SSCM in an RCT in a new larger sample.

The subsidiary aim of the study is to explore mediators and moderators of treatment outcome.

## Methods/Design

### Hypotheses

(1) A specific, empirically-based treatment model focusing on four core maintenance factors (MANTRA) will be superior to SSCM in producing greater weight gain and greater improvement in eating-disorder related psychopathology in adults with AN at six and twelve months.

(2) MANTRA will be more cost-effective than SSCM, showing lower costs at six and twelve months. Specifically, it will be associated with fewer and shorter hospitalizations during treatment and follow-up compared to SSCM.

### Research plan

#### Trial design

This is a multi-center two-arm superiority trial which will evaluate the efficacy and cost-effectiveness of MANTRA compared to SSCM, in consecutive referrals of adult outpatients with AN. Patients will be randomly allocated to either MANTRA or SSCM. More detail regarding the randomization procedure is provided below. Patients in both groups will be offered the same amount of therapy.

**Figure 1:**
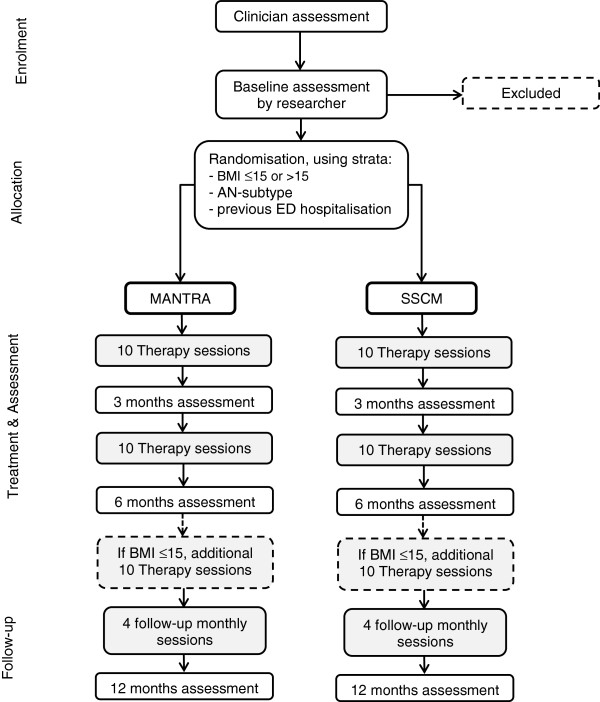
Study design.

The study design is shown in Figure 1.

Outcomes will be measured pre-randomization, at six months (that is, around the end of weekly treatment sessions) and at twelve months follow-up. In addition, mediators of treatment outcome will be assessed mid treatment (three months). Post treatment outcomes will be assessed by researchers who are not involved in the treatment process and steps are undertaken to ensure that researchers remain blind to the patient’s treatment group. Every effort will be made to include patients who drop out of treatment in the follow-up assessments to enable intention-to-treat analysis.

#### Ethical approval

Ethical approval for the MOSAIC Trial has been obtained from Central London REC 4, National Research Ethics Service, Royal Free Hospital, London, NHS REC Reference: 10/H0714/9. Participants are not exposed to risk, receive verbal and written information before starting and informed written consent is obtained from participants before they enter the study. The research was conducted in compliance with the Helsinki Declaration.

### Interventions

#### Commonalities between both treatments

In both treatments, patients will receive 20 once-weekly individual sessions of therapy together with four follow-up sessions, with monthly spacing. In low-weight patients with a BMI of ≤15 kg/m^2^, weekly treatment will be extended up to 30 sessions plus four follow-ups. In both treatments two additional sessions with a close other will be offered as well as an assessment from the team’s dietician with follow-up dietetic sessions as needed. Ongoing monitoring of physical risk is an integral part of both treatments. Therapy sessions will last approximately 50 minutes, however in SSCM, from the middle stage of treatment session duration may be reduced to 30 minutes at the therapist’s discretion, as outlined in the original SSCM manual (McIntosh, Jordan, Joyce, McKenzie, Luty, Carter, and Bulik, unpublished). Whilst this means that SSCM patients in our trial will potentially receive somewhat less therapy time than those allocated to MANTRA, we thought that following the original SSCM protocol has the advantage that our study will be comparable to other trials using SSCM.

#### MANTRA

The MANTRA model [5] proposes that four factors, linked to underlying obsessional and anxious/avoidant personality traits, are central to the maintenance of AN and need to be addressed in treatment.

These are:

(a) a thinking style characterized by inflexibility, attention to detail at the expense of the bigger picture and fear of making mistakes;

(b) impairments in the socio-emotional domain (such as avoidance of the experience and expression of emotions and the socio-emotional triggers that arouse them);

(c) pro-anorexia beliefs (that is, beliefs about the utility of AN in helping the person manage their life). These are one aspect of a broader set of illness beliefs and it is the interaction between beliefs about the illness and other non-illness related beliefs (that is, beliefs about self, others and the world) that gives the illness its own unique meaning for a particular person. Examples of typical pro-AN beliefs include: AN keeps me safe, AN numbs my emotions, AN helps me to express my distress [21-23];

(d) the response of close others, including anxiety, worry, blame, criticism, or hostility.

Thus it is hypothesized that MANTRA changes these four putative mediating factors which in turn improve the clinical outcomes (eating disorder symptoms).

MANTRA treatment is centered around a patient-manual (see data supplement DS1 in Schmidt *et al*. [19] for details), the use of which is tailored to the needs of the individual. The therapist style is that of motivational interviewing [24], that is, reflective, responsive and collaborative. Based on an in-depth physical, psychological, neuropsychological and socio-emotional/relational assessment a collaborative case formulation is developed, which is trait focused and builds on people’s strengths. Feedback, for example, about medical risk, and thinking style is used to increase motivation to change. The principles of behavioral change are used to guide people towards recovery [25,26]. There is a clear hierarchy of treatment procedures depending on the person’s clinical profile and balancing treatment motivation, level of medical risk, and personal resources and supports available. Close others are invited to participate flexibly in sessions as necessary.

#### SSCM

This treatment was developed as a comparison treatment in an RCT comparing CBT, IPT and SSCM [18,27]. SSCM is designed to be delivered by health professionals trained in the treatment of eating disorders and aims ‘*to mimic outpatient treatment that could be offered to individuals with AN in usual clinical practice’*. This treatment links features of clinical management and supportive psychotherapy. The former emphasizes the therapist’s expertise in providing safe and appropriate patient management and care, including education and support. The latter emphasizes the therapist’s acceptance of the patient and a hopeful, positive stance; a focus on the patient’s strengths, a collaborative, reflective conversational style, using praise, reassurance and advice as appropriate. The abnormal nutritional status and dietary patterns of AN are seen as central to SSCM. The treatment emphasizes the resumption of normal eating and restoration of weight and provides information on weight gain and weight maintenance strategies, energy requirements and relearning to eat normally. The remaining therapy content is determined by the patient. SSCM for AN has three phases. Early on, the patient is oriented towards the treatment, target symptoms are identified and goals for weight gain and normalizing eating are agreed upon. In the middle phase, target symptoms are monitored and the patient is supported and given encouragement in their attainment of the dual goals of weight restoration and normal eating. In the final phase, issues related to the ending of therapy are discussed, including plans for the future and the end of the therapeutic relationship. Further details of this treatment are described in McIntosh *et al*. [28]. There is also a manual for therapists (McIntosh, Jordan, Joyce, McKenzie, Luty, Carter, and Bulik, unpublished) which contains psycho-educational handouts for patients on topics such as the ineffectiveness of laxatives and the impact of societal beliefs about body shape and weight that are used flexibly throughout treatment.

#### Therapist training and supervision, treatment fidelity, untoward events and protocol adherence

All therapists will be experienced eating disorder therapists. We will use principles for enhancing treatment fidelity outlined by the NIH Behavior Change Consortium [29]. All therapists will attend two initial training days on MANTRA and SSCM and over the course of the study, update ‘booster’ training days will be held at regular intervals to avoid ‘therapeutic drift’. All therapists will see patients in both conditions. Such a ‘crossed design’ allows for within-therapist assessment of intervention effects and avoids standard error inflation by general therapist effects (see statistical analysis section on how general and therapy-specific therapist effects are modeled). Regular weekly supervision will be provided to therapists by senior clinicians in their team and separately for the two treatment conditions, to avoid contamination across therapies. It is planned that each therapist will see eight or more patients. Patients will be allocated to therapists based on therapist availability. To ensure competent and uniform treatment delivery, psychotherapy sessions will be audiotaped and a random selection of three audiotapes per patient will be reviewed for adherence to the two treatments. An adaptation of the Collaborative Study Psychotherapy Rating Scale will be used to assess whether the two treatments can be reliably distinguished [27]. In addition, we will use the Motivational Interviewing Treatment Integrity (MITI version 2) rating scale [30], and components of the second version of the Motivational Interviewing Skill Code (MISC version 2) [31]. These motivational measures are included as the style of MANTRA is explicitly motivational and that of SSCM has implicit elements of motivational interviewing.

Therapists will keep a case record form for each of their trial patients on which they record each session, briefly describe the session content, session duration, and who attended (including close others involved in the session), note the patient’s eating disorder symptoms, and record any untoward events, according to pre-specified criteria. Any protocol violations, for example, caused by the patient’s admission to hospital will also be recorded here. Criteria for an admission to hospital will be the same as described below under exclusion criteria.

### Inclusion/exclusion criteria

#### Inclusion criteria

Consecutive patients referred to the specialist eating disorder service by their GP will be offered participation if they are:

(a) aged between 18 and 60 years;

(b) have a BMI of 18.5 or below;

(c) have a DSM-IV diagnosis of AN or Eating Disorder Not Otherwise Specified (EDNOS). Our definition of EDNOS is based on that by Thomas *et al*. [32] and includes people who fulfill all criteria of AN, except the weight criterion; those who fulfill all criteria for AN but still have menses; those without a fat phobia; and those with partial AN (defined as having features of AN but missing at least two of the four diagnostic criteria).

The decision to include EDNOS patients with a BMI cut-off of 18.5 kg/m^2^ is supported by a recent large meta-analysis of EDNOS which suggests that AN with a more lenient weight criterion and without amenorrhea is very similar to AN as defined currently [32]. We chose a BMI cut-off of 18.5 as this is the WHO cut-off for being underweight. Additionally, this BMI criterion was also used in our previous study and in another large recent AN trial [19,33].

#### Exclusion criteria

(a) life-threatening AN requiring immediate inpatient treatment as defined in the UK NICE guidelines for eating disorders [17];

(b) insufficient knowledge of English to understand the treatment; learning disability; severe mental or physical illness which needs treatment in its own right (for example, psychosis or diabetes mellitus); substance dependence or pregnancy.

We will not exclude patients on antidepressants, provided they are on a stable dose, that is, for at least four weeks.

### Outcome measures

All outcome measures will be collected at baseline, six and twelve months, except the treatment credibility/acceptability visual analogue scale (VAS) which will be collected only at six and twelve months. Potential mediators of treatment outcome will also be collected at three months (midtreatment).

#### Primary outcome

•Body Mass Index (kg/m^2^) at twelve months.

#### Secondary outcomes

•Body Mass Index (kg/m^2^) at six months.

•Eating Disorders Examination (EDE) global and subscale scores [34]. The EDE is a widely used, semi-structured interview that generates four subscale scores: dietary restraint, eating concern, weight concern and shape concern. The mean of these four subscales is used to create a global score. For patients unwilling/unable to do the EDE interview, the questionnaire form of this assessment (EDE-Q) will be used instead. The EDE-Q has been found to have similar validity to the EDE interview [35].

##### Other psychopathology

The Depression, Anxiety and Stress Scale - 21 (DASS-21) [36], Obsessive Compulsive Inventory (OCI) [37].

##### Potential mediators

The Cognitive Flexibility Scale [38], Beliefs about Emotions Scale [39], The Emotion Regulation Questionnaire [40] and a visual analog scale (VAS) assessing motivation and social support.

#### Treatment credibility/acceptability

•Visual analog scales (VAS) of credibility and acceptability of treatment, and use of what they have learnt in treatment.

#### Neurocognitive and social-cognitive measures (also potential moderators)

•The Wisconsin Card Sorting Task [41,42] assesses cognitive flexibility (or set-shifting ability). It is a commonly used task and involves matching stimulus cards with one of four category cards. The stimuli are multidimensional according to color, shape and number.

•The Brixton Spatial Anticipation Task [43]. This also measures set-shifting ability. Participants predict the movement of a blue circle across 10 different positions, adapting their predictions as the pattern of movement changes.

•The Rey-Osterrieth Complex Figure Test [44,45]. This is a test of central coherence and evaluates ability to plan, organize and assemble complex information. Participants are asked to copy a complex figure design.

•Baron-Cohen’s ‘Reading the Mind in Film’ task [46]. This measures complex Theory of Mind and consists of viewing 22 brief film clips, after which participants are asked to choose which of four words best describes how the given character was feeling at the end of the scene.

#### Costs and psychosocial impairment

•The Client Services Receipt Interview (CSRI) [47]. This is a self-report inventory of service use which facilitates estimation of support costs. It will be adapted for the current study to cover a wide variety of hospital, mental health, and community-based services as well as medications, impact of employment and additional personal expenditure due to the eating disorder.

•The Clinical Impairment Assessment (CIA) [48]. This is a self-report measure of global psychosocial impairment resulting from the individual’s eating disorder behaviors.

### Randomization

The generation and implementation of the randomization sequence is conducted independently from the trial team by the King’s Clinical Trials Unit (CTU). Once the initial assessment has been carried out and the patient recruited to the trial, the researcher enters patient ID and stratifier details into the web-based CTU system. Patients are then allocated to one of the two trial arms using a restricted stratified randomization algorithm. The strata will be (1) severity of weight loss (BMI below or above 15), (2) AN-subtype (restricting or binge/purge) and (3) previous admission within an eating disorder inpatient unit, as these factors are known to affect treatment outcome and rates of possible future hospitalization. The stratification will be implemented by minimized randomization with a random component. The first N cases (N will not be disclosed) will be allocated entirely at random to further enhance allocation concealment.

### Blinding and methods for protecting against other sources of bias

It is not possible for patients or therapists to be blind to the type of treatment. The research assessor, however, will be blind to treatment allocation. In order to test whether the researcher has remained blind to the treatment allocation, they will be required to make a judgment at the end of the twelve months assessment as to which treatment they believe the person has received.

### Sample size

We observed a mean weight gain of 7.3 kg (standard deviation 4 kg) in an unpublished series of nine pilot patients treated with MANTRA. The mean weight gain for SSCM was previously estimated as 4 kg (McIntosh *et al*. [18]). We derived a conservative estimate of the group difference by a low estimate of the weight gain under MANTRA (mean – 0.8 × standard error = 6.5 kg) minus the weight gain estimate for SSCM; giving a difference of 2.5 kg. A sample size of n = 55 per group will have 90% power to detect a difference in mean weight gain of 2.5 kg assuming a common weight gain standard deviation of 4 kg (as per unpublished series of the nine pilot patients) and using an independent samples *t*-test with a significance level of alpha = 0.05. Correcting for 20% attrition (as found in our previous studies), a total of 138 patients will be needed. (The sample size calculation was not inflated for therapist effects because therapists were crossed with treatments, which allows us to take account of general therapist effects in the analysis, and treatment-specific therapist effects were thought to be negligible).

### Recruitment

Patients will be recruited from several centers: South London and Maudsley NHS Foundation Trust (catchment area: two million); North East London Foundation Trust Eating Disorders Service (catchment area 650,000); Barnet, Enfield & Haringey Mental Health NHS Trust (catchment area five million); Oxford Health NHS Foundation Trust (catchment area 1.1 million for Oxfordshire and Buckinghamshire).

### Data management

Data will be checked for entry errors by performing double data entry for 10% of the data. Quality of the data will be tested by examining the data for impossible values by looking at data ranges. No post treatment data will be released until the database is locked. Statistical analysis will be blind to treatment arm.

### Statistical analysis

Outcomes will be analyzed on an intention-to-treat (ITT) basis, that is, participants will be analyzed in the group to which they were randomized irrespective of their compliance with the assigned scheme.

#### Primary outcome analysis

BMI at pretreatment (baseline), posttreatment (six months) and follow-up (twelve months) will be analyzed using a linear mixed effects model. In this model, the explanatory variables with fixed effects are treatment (MANTRA or SSCM), time (six months or twelve months), the treatment x time interaction, baseline BMI and randomization stratifiers (severity of weight loss, AN-subtype, and previous hospitalization). Correlation due to seeing the same therapist will be modeled by random effects that vary at the level of the therapist (accounting for general therapist effects) and a second set of random effects that vary at the level of the therapist within a specific treatment (a therapist x treatment interaction accounting for therapy-specific therapist effects). Correlation due to repeated measures will be accounted for by subject-varying random effects. Models will be fitted using maximum likelihood [49]. Model fitting will produce treatment effects estimates at posttreatment (secondary BMI outcome) and twelve months (primary BMI outcome). In the case of missing values in posttreatment BMI, the analysis is valid under the missing at random (MAR) assumption which stipulates that missingness is only driven by variables included in the mixed model. We will summarize the relation between demographic and clinical variables at baseline and study drop-out by twelve months (that is, those lost to follow-up or who actively withdrew from the study). Any baseline variables found to be predictive of missingness will be included as further explanatory variables in the mixed model.

Adherence with allocated treatment will be measured by the number of sessions attended and patients classified as ‘MANTRA completers’, ‘SSCM completers’ or ‘non-completers. Treatment completion will be defined as attending a minimum of 15 out of 20 weekly sessions [50]. In the case of non-adherence with randomized treatments, the ITT estimate no longer provides an efficacy assessment (only effectiveness under the current setting) and we will estimate the outcome difference between those receiving SSCM and MANTRA (relative efficacy, or more specifically the complier average causal effects or CACE) using instrumental variables methods (see for example, Dunn *et al*. [51]).

#### Secondary outcome analyses

Secondary outcome variables are all continuous and will be analyzed in the same way as the BMI variables. There are a considerable number of assessments of treatment effects on secondary outcomes. Thus interpretation of these treatment effect estimates will need to take into account the impact of multiple inferences.

##### Exploratory mediation assessment

Four of the secondary outcomes were chosen since they measure the core maintenance factors targeted by the MANTRA intervention. In other words these variables are putative mediators of the effect of MANTRA on patient ED outcomes. We will use the Baron and Kenny regression approach [52] to assess the potential of each of these four variables as mediators of patient ED outcomes (BMI and EDE global score). Such analyses make restrictive assumptions; the most important being that there is no hidden confounding of the effect of the mediator on the outcome. Thus we only use this as an exploratory approach to empirically re-formulate our basic (theoretical) MANTRA process model. The parameters of this model will yet have to be estimated without bias from future studies that have been designed for mediation analysis.

##### Exploratory moderation assessment

The study was not powered to detect treatment effect modification by baseline variables. We will carry out exploratory moderator analyses by including interactions between treatment and putative moderators (see potential moderators’ list above) in respective linear mixed models. The results will suggest moderation hypotheses to be investigated in future trials.

All analyses will be carried out in Stata 12 [53].

#### Economic analysis

Service use patterns will be described and service costs calculated using a well-established compendium of unit costs [54] or those specifically estimated for this study, using a comparable estimation method. Use of MANTRA and SSCM will be recorded by therapists on the case record form noting the number of sessions each participant attended, the duration of each session and details of staff involved in providing the intervention. From these data, the cost of the intervention will be estimated.

Cost-effectiveness analysis will then be conducted for the period from baseline to twelve months follow-up using the primary outcome measures, and the CIA.

### Reporting of trial

The trial data will be reported in line with the extension of CONSORT guidance for trials assessing non-pharmacological treatments [55,56].

## Discussion

The aim of the MOSAIC study is to compare the efficacy, cost and cost-effectiveness of two manual-based treatments, MANTRA and SSCM, in adult outpatients with AN and EDNOS-AN. The secondary aim is to explore mediators and potential moderators of treatment outcome.

Psychotherapeutic interventions are the treatment of choice for AN and related conditions. However, the evidence-base for such treatments of adults with AN is extremely limited. A number of small trials have tested a range of therapies, yet there is no clear front runner. There is an urgent need to develop more efficacious treatments for adults with AN. Previous psychosocial treatments for AN have been adapted from those for other disorders and the effectiveness of a tailor-made intervention is yet to be tested. MANTRA is a treatment based on a specific maintenance model of AN. It draws on neuropsychological, social-cognitive and personality trait research in AN and includes both intra- and interpersonal maintaining factors. It also has some preliminary evidence for acceptability and effectiveness among the AN patient group [19,20].

The comparison treatment, SSCM, has been selected as it was found to be superior to two other active treatments [18] and is therefore a suitable alternative to MANTRA. However, the advantages of SSCM appear to fade over time [50]. Therefore longer-term follow-up is needed to determine whether treatment effects of SSCM and MANTRA persist over time, which we plan to explore in future studies.

### Potential implications

The MOSAIC study is the first large-scale RCT to compare the efficacy of MANTRA and SSCM for adult outpatients with AN or EDNOS-AN. The economic analysis will give insight into the total cost of treating the disorder and inform future best practice guidance. The results of this study will provide a rigorous evaluation of a novel treatment for AN (MANTRA) which could be implemented in the NHS and made available to services across the UK.

### Strengths

This trial has a number of strengths. In particular the large sample size provides sufficient power to draw meaningful conclusions. Generalizability of results is facilitated by maximizing recruitment; four separate sites participate in the study and the broad inclusion criteria ensure inclusion of patients along the full spectrum of AN severity. Therapist bias is reduced as all therapists treat patients using both MANTRA and SSCM, and research bias is reduced by researcher blinding to treatment and stratified randomization. To help prevent trial drop-out, treatment and research are kept separate.

### Challenges

Maintaining motivation over the prolonged study period may be challenging. All clinical and research personnel involved in MOSAIC will receive a monthly newsletter providing updates on recruitment, answering queries, and offering encouragement to each NHS site. There will also be a facility to allow anonymous feedback from clinicians about their experience of the two interventions and of taking part in an RCT. Quality control measures have also been considered. To avoid therapeutic drift, clinicians will receive ‘booster’ training days over the course of the study and work using separate treatment manuals. Therapists will attend weekly supervision by senior clinicians. Supervision for MANTRA and SSCM will be carried out separately to avoid contamination across therapies. Psychotherapeutic sessions will be audio taped and case record forms will be kept for individual patients to ensure adherence to treatment protocol and the uniformity of treatment delivery. To aid in participant retention we will keep a certain level of continuity in the researcher-participant pairing and collect alternative contact information should participant circumstances change. Through understanding that research assessments may seem arduous at times, we aim to accommodate participants wherever possible by offering home visits, telephone interviewing and financial reimbursement.

## Conclusion

To conclude, this paper sets out a protocol for a multi-center RCT that will enhance the current knowledge of the relative efficacy of potential treatments for AN, using a number of neuropsychological, social cognitive and psychological measures.

## Trial status

Recruitment for the trial is on-going. Data collection will continue until end of 2013.

## Abbreviations

AN: Anorexia nervosa; BMI: Body mass index; CACE: Complier average causal effects; CBT: Cognitive behavioral therapy; CIA: Clinical Impairment Assessment; CSRI: Client Services Receipt Interview; CTU: Clinical trials unit; DASS-21: Depression Anxiety, Stress Scale – 21; DSM-IV: Diagnostic and Statistical Manual of mental disorders; EDE: Eating disorder examination; EDEQ: Eating disorder examination questionnaire; EDNOS: Eating disorder not otherwise specified; EDNOS-AN: Eating disorder not otherwise specified anorexia nervosa type; IPT: Interpersonal psychotherapy; MANTRA: Maudsley Model of Treatment for Adults with Anorexia Nervosa; MAR: Missing at random; MHRN: Mental Health Research Network; MISC: Motivational Interviewing Skills Code; MITI: Motivational Interviewing Treatment Integrity; MOSAIC: Maudsley Outpatient Study of Treatments for Anorexia Nervosa and Related Conditions; NART: National Adult Reading Test; NICE: National Institute for Health and Clinical Excellence; NIH: National Institute of Health; OCI: Obsessive compulsive inventory; RCT: Randomized controlled trial; SSCM: Specialist supportive clinical management; VAS: Visual analogue scale; WHO: World Health Organization

## Competing interests

The authors declare that they have no competing interests.

## Authors’ contributions

US is the principal investigator of the study. LS, LR, EJ-S, NB and LW are co-investigators. SL is the leading biostatistician. JB is the lead in providing health economics for the study. US, JT and SL conceived the study and made substantial contributions to the concept development, design, MANTRA manual and methodology. US and SL drafted the original version of the study protocol. BR and AL assisted in the preparation of the study protocol. BR, AL, MK, HDJ, HB, RL, CW and SG collected data and assisted in coordination of the project. All authors read and approved the final manuscript.
